# Supplementary material for: The Identity of the Constriction Region of the Ribosomal Exit Tunnel Is Important to Maintain Gene Expression in Escherichia coli
Source: Microbiol Spectr. 2022 Mar 21;10(2):e02261-21. doi: 10.1128/spectrum.02261-21 (PMC9045200; doi:10.1128/spectrum.02261-21)
Supplement: SUPPLEMENTAL FILE 2 — Supplemental material. Download SPECTRUM02261-21_Supp_2_seq13.pdf, PDF file, 1.9 MB [file spectrum02261-21_supp_2_seq13.pdf]

SUPPLEMENTARY DATA

Table S1. List of strains and plasmids used in this study

Strains	Genotype or Description	Source or Reference
SVS1144	W3110 *bglR551 Δ(lac-argF)U169* λ *tnaP tnaC-UGA tnaA'-'lacZ*	[1]
SM1110	*araΔ(lac pro)thr trpam supCts htpRam165/F' lacI^{Q} lac^{+} pro^{+}s10::Km^{r} clpA clpX/ pS10*	[2]
SMCVF-1	SVS1144 s10::Km^{r} /pS10	This work
SMCVF-2	SVS1144 s10::Km^{r} / pS10 *rplV* (K90D)	This work
SMCVF-3	SVS1144 s10::Km^{r} / pS10 *rplD* (R61D)	This work
SMCVF-4	SVS1144 s10::Km^{r} / pS10 *rplV* (K90D) *rplD* (R61D)	This work
CY15000	F-, *λ*$^{-}$, *IN(rrnD-rrnE)1*, *rph-1*, *tnaA5*	[3]
**Plasmids**		
pS10	ColA derivative S10 operon; Kan^{R}	[2]
pLP8	P_{lac}*lacI^{q}*Kan^{R}	[4]
pTnaA	pLP8 derivative *tnaA;* Kan^{R}	[5]

Table S2. List of primers used in this study to mutagenize the proteins uL4 and uL22 produced from the pS10 plasmid

Gene Mutation	Primer Sequence (5' - 3')
rplV	
K90D	F: CGCATTATGCCGCGTGCA**GAC**GGTCGTGCAGATCGCATC<br>R: GATGCGATCTGCACGACC**GTC**TGCACGCGGCATAATGCG
rplD	
R61D	F: CGGTAAAAAACCGTGG**GAC**CAGAAAGGCACCGGC<br>R: GCCGGTGCCTTTCTG**GTC**CCACGGTTTTTTACCG

Table S3. Minimum inhibitory concentrations (MIC) of *E. coli* cells expressing wild type uL22 or uL22(K90D) mutant proteins to several antibiotics.

**uL22**	**MIC (µg/ml)**[1]								
	**QUI**	**VIR**	**CLI**	**ERY**	**AZI**	**TEL**	**CAM**	**SPC**	**AMP**
**WT**	>512	>256	>1024	**128**	**64**	**64**	1	32	4
**K90D**	>512	>256	>1024	**256**	**128**	**256**	1	32	4

[1] MIC: Minimal Inhibitory Concentration (expressed in µg/mL); QUI: Quinupristin (a streptogramin); VIR: Virginiamycin (a streptogramin); CLI: Clindamycin (a lincosamide); ERY: Erythromycin (a macrolide); AZI: Azithromycin (a macrolide); TEL: Telithromycin (a ketolide); CAM: Chloramphenicol (a phenicol); SPC: Spectinomycin (an aminoglycoside) ; AMP: Ampicillin (cell wall synthesis inhibitor). This table represents the values of at least three independent experiments.

Cells were grown overnight in LB media, diluted, and added to the appropriate well in a 96-well plate with prepared concentrations of the specified antibiotic. These cells were incubated with shaking in a microplate reader (SpectraMax iD5) for 12 hours at 37˚C and the OD_{600} was measured periodically. MIC values correspond to the antibiotic concentrations where the OD_{600} absorbance values detected at any time during the period of incubation were not different from the starting OD_{600} absorbance values.

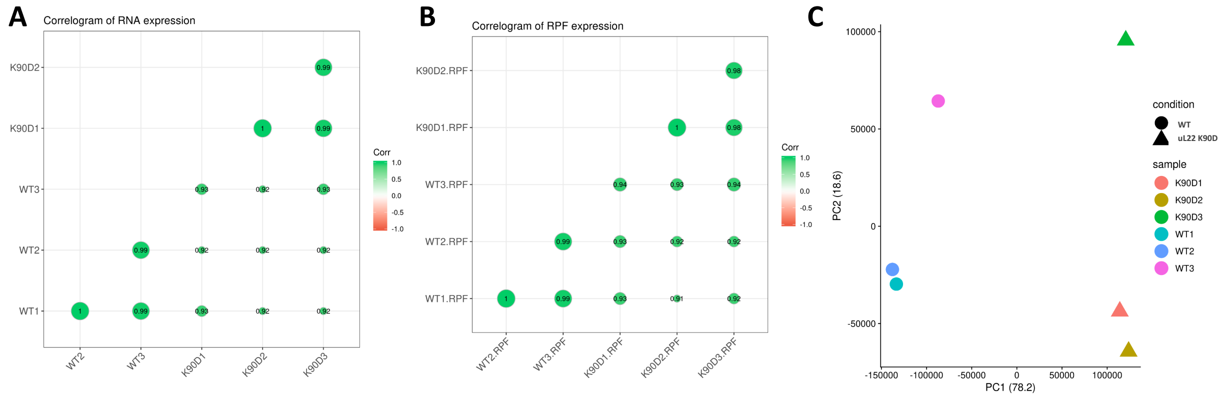

Figure S1. Reproducibility between ribosome profiling samples and correlation of RPFs to mRNA levels. (A) Correlograms of normalized RNA and **(B)** RPFs values between all samples. **(C)** Principal component (PC) analysis among different samples in two groups. For PC1 samples group according to the cell strain samples, and PC2 groupings correspond to different harvest dates of the samples. These groupings indicate true differences between the wild type and uL22 K90D cells.

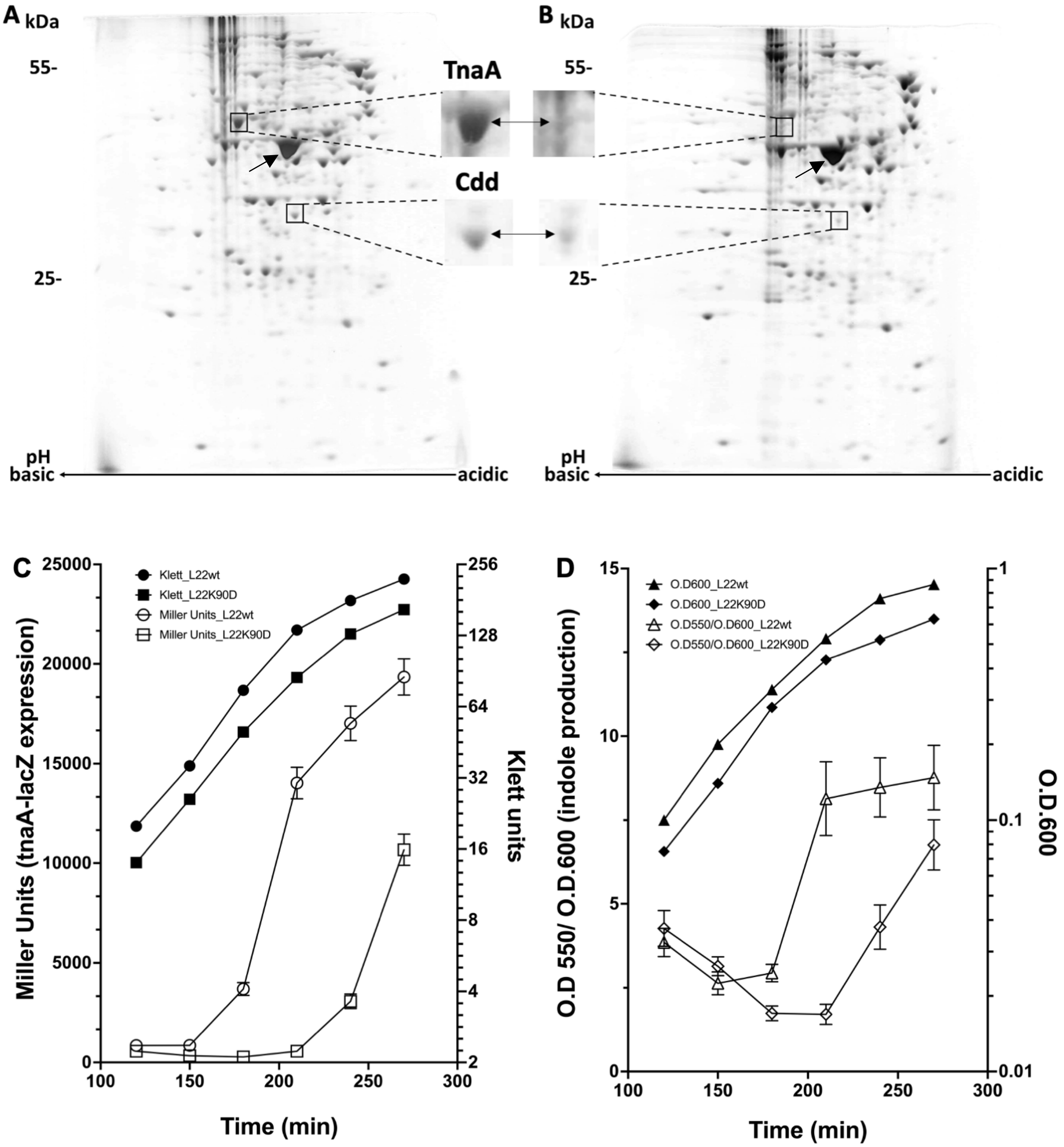

Figure S2. **Analysis of the expression of the tryptophanase and TnaA-LacZ reporter in *E. coli* cells harboring the wild type uL22 and K90D mutant. A) and B)** Standard 2-D gel electrophoresis of cells containing **(A)** wild type **(B)** mutant protein uL22(K90D) were performed as previously described [6]. Cultures grown to approximately an $O.D._{600}$ of 0.6 were harvested by centrifugation and cells were processed for protein extraction. Proteins were visualized using Coomassie Brilliant Blue R-250 stain. Protein spots of interest were cut from gels and protein identities were determined using mass spectrophotometry. Zoomed view of tryptophanase (TnaA) and Cdd protein spots are shown. The intensities of the TnaA and Cdd spots as well as the protein spot indicated with an arrow (reference) were quantified using particle analysis provided by the Fiji-image processor. For each gel, the TnaA and Cdd intensities were normalized with respect to the reference. Comparison of the TnaA and Cdd spot intensities between both gels were obtained by dividing the normalized intensity of the TnaA and Cdd protein spots from the K90D strain gels with by the corresponding spots from the L22 wild type strain gels. We have

determined that for these two gels the TnaA and Cdd spots seen in the L22 K90D sample are 8% and 53% to those seen in the the L22 wild type sample. **C)** Expression of the *tnaA-lacZ* reporter gene of both strains was determined by calculating the activity in Miller units of the β-Galactosidase enzyme. **D)** Indole production was determined by mixing 100 μl of culture with 30 μl of Kovack's reagent. After 5 min, the mixtures were centrifuged for 2 min at 10,000 g and the liquid overlayer was taken for reading using a microplate reader. Generation of quinidal red-violet complex, product of the reaction of indole with p-dimethylaminobenzaldehide, was determined by absorption at $O.D._{550}$. Absorption values were normalized with respect cellular density determined using absorption at $O.D._{600}$. Samples used to produce both plots, **(C)** and **(D)** were taken at different time points of cell growth cultures grown in LB at 37°C. An example of a growth curve is shown in each plot. Klett units and OD_{600} values from the same cultures are used as references. Error bars indicate mean ±SD (n=5).

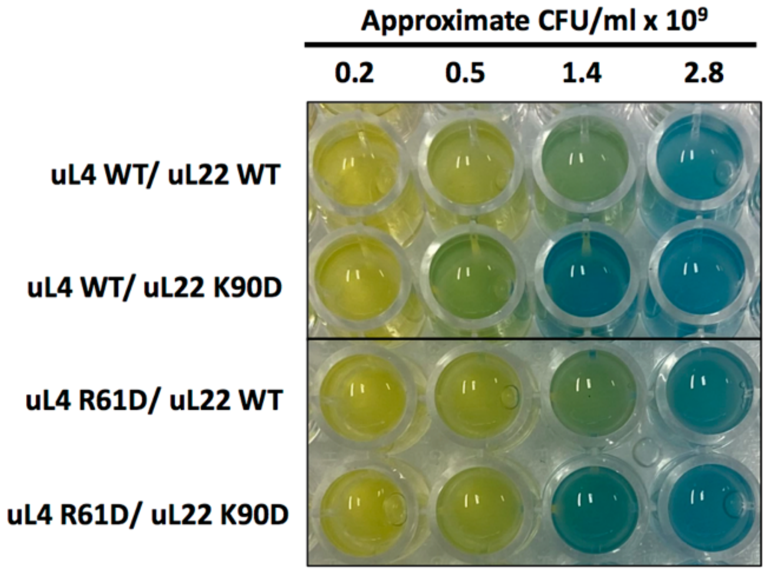

Figure S3: Glutamate decarboxylase (GAD) activity of the single uL4(R61D) or single uL22(K90D) mutant and their double mutant strains. The uL22(K90D) and uL4(R61D)/uL22(K90D) exhibit significantly higher GAD activity relative to the WT parental strain and the uL4(R61D) single mutant.

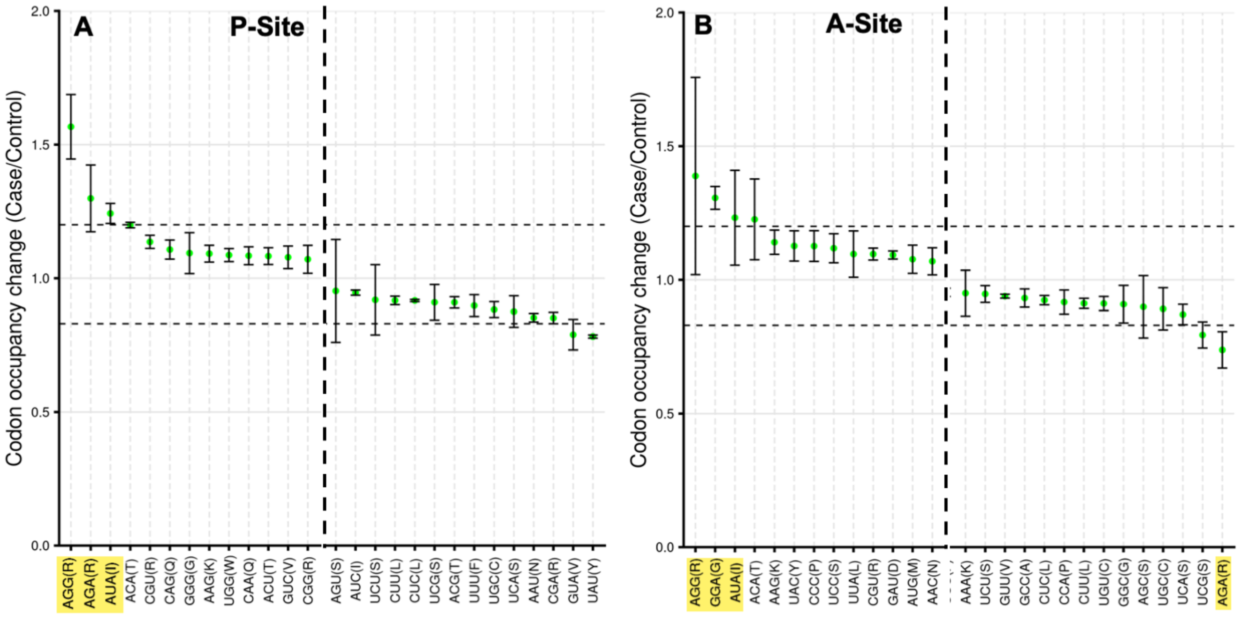

Figure S4: Changes in the codon occupancy at the P-site (panel A), and A-site (panel B) of the uL22(K90D) strain. Ribosome occupancy values (case/control ratios) were obtained from the uL22(K90D) strain (case) relative to the wild type L22 strain (control). Standard deviations are shown for each codon obtained from 3 biological replicates for each L22(WT) and L22(K90D) strains . Codons highlighted in yellow show the mean changes beyond the established threshold. The RiboToolkit codonstat tool was used to examine specific codon frequency in genes indicated as having altered translation efficiency based off of A- and P-site codon occupancy indicated in the group analysis, background setting was changed to random-select and codons to all codon.

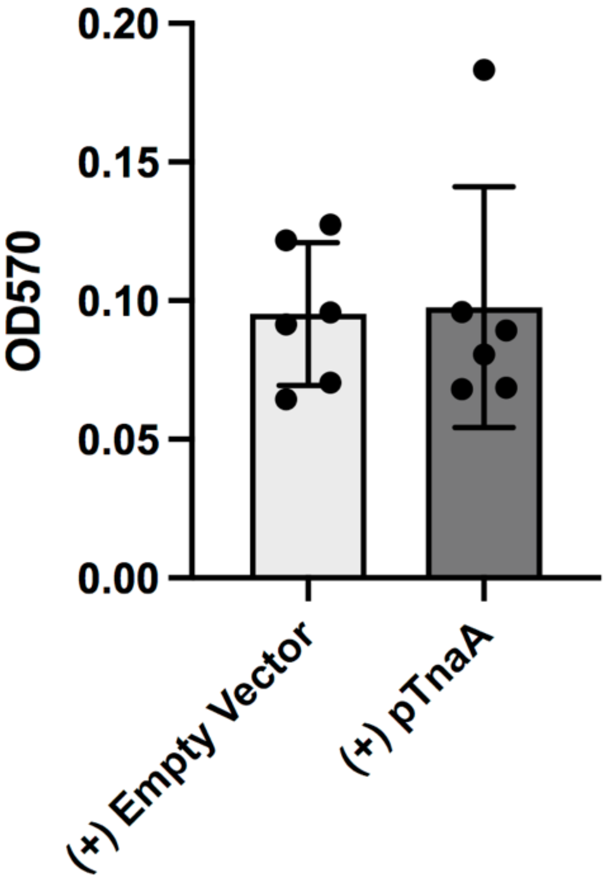

Figure S5. **Biofilm formation in the presence or absence of tryptophanase in *E. coli* CY15000.** An isogenic *tnaA* mutant (CY15000) was transformed with either pLP8 (empty vector) or pTnaA (pLP8 with *tnaA*) and biofilm formation of the resulting strains was measured. Results revealed that CY15000 biofilm formation is not affected by the overexpression of *tnaA*. Error bars represent standard deviation (N=6).

REFERENCES

[1] Stewart, V., and Yanofsky, C. (1985). Evidence for transcription antitermination control of tryptophanase operon expression in *Escherichia coli* K-12. *J. Bacteriol.*, **164**, 731–40.

[2] Moore, S. D., Baker, T. A., and Sauer, R. T. (2008). Forced extraction of targeted components from complex macromolecular assemblies. *Proc. Natl. Acad. Sci. U. S. A.*, **105**, 11685–11690.

[3] Yanofsky, C., and Horn, V. (1981). Rifampin resistance mutations that alter the efficiency of transcription termination at the tryptophan operon attenuator. *J. Bacteriol.*, **145**, 1334–1341.

[4] Potluri, L., Karczmarek, A., Verheul, J., Piette, A., Wilkin, J. M., Werth, N., Banzhaf, M., Vollmer, W., Young, K. D., Nguyen-Disteche, M., and den Blaauwen, T. (2010). Septal and lateral wall localization of PBP5, the major D,D-carboxypeptidase of *Escherichia coli*, requires substrate recognition and membrane attachment. *Mol. Microbiol.*, **77**, 300–323,.

[5] Li, G., and Young, K. D. (2012). Isolation and identification of new inner membrane-associated proteins that localize to cell poles in *Escherichia coli*. *Mol. Microbiol.*, **84**, 276–295.

[6] O'Farrell, P. H. (1975). High resolution two-dimensional electrophoresis of proteins. *J. Biol. Chem.*, **250**, 4007–4021.
